# Gait Synergy Analysis and Modeling on Amputees and Stroke Patients for Lower Limb Assistive Devices

**DOI:** 10.3390/s22134814

**Published:** 2022-06-25

**Authors:** Feng-Yan Liang, Fei Gao, Junyi Cao, Sheung-Wai Law, Wei-Hsin Liao

**Affiliations:** 1 Department of Biomedical Engineering, The Chinese University of Hong Kong, Shatin, Hong Kong, China; fyliang@hainanu.edu.cn; 2 Key Laboratory of Biomedical Engineering of Hainan Province, School of Biomedical Engineering, Hainan University, Haikou 570228, China; 3 Department of Mechanical and Automation Engineering, The Chinese University of Hong Kong, Shatin, Hong Kong, China; fei.gao@siat.ac.cn; 4 Shenzhen Institute of Advanced Technology, Chinese Academy of Sciences, Shenzhen 518055, China; 5 School of Mechanical Engineering, Xi’an Jiaotong University, Xi’an 710049, China; caojy@xjtu.edu.cn; 6 Tai Po Hospital, Hong Kong, China; lawsw@cuhk.edu.hk

**Keywords:** synergy, gait, stroke, amputee, LSTM, wearable robot

## Abstract

The concept of synergy has drawn attention and been applied to lower limb assistive devices such as exoskeletons and prostheses for improving human–machine interaction. A better understanding of the influence of gait kinematics on synergies and a better synergy-modeling method are important for device design and improvement. To this end, gait data from healthy, amputee, and stroke subjects were collected. First, continuous relative phase (CRP) was used to quantify their synergies and explore the influence of kinematics. Second, long short-term memory (LSTM) and principal component analysis (PCA) were adopted to model interlimb synergy and intralimb synergy, respectively. The results indicate that the limited hip and knee range of motions (RoMs) in stroke patients and amputees significantly influence their synergies in different ways. In interlimb synergy modeling, LSTM (RMSE: 0.798° (hip) and 1.963° (knee)) has lower errors than PCA (RMSE: 5.050° (hip) and 10.353° (knee)), which is frequently used in the literature. Further, in intralimb synergy modeling, LSTM (RMSE: 3.894°) enables better synergy modeling than PCA (RMSE: 10.312°). In conclusion, stroke patients and amputees perform different compensatory mechanisms to adapt to new interlimb and intralimb synergies different from healthy people. LSTM has better synergy modeling and shows a promise for generating trajectories in line with the wearer’s motion for lower limb assistive devices.

## 1. Introduction

Nowadays, lower limb assistive devices are designed to help subjects with motor impairments, such as amputees and stroke patients, restore locomotion function. Significant achievements have been made for lower limb exoskeletons and active prostheses such as HAL [1], Lokomat [2], powered ankle–foot prostheses [3] and powered transfemoral prostheses [4]. In our group, we have developed an active lower limb exoskeleton [5], knee brace [6], and ankle–foot prosthesis [7] to help individuals who have lost mobility or suffered from pathological gait restore/regain locomotion function. Active assistive devices were designed to help the injured joints or replace the amputated joints to reproduce the required function. To achieve that, these assistive devices need to identify the intention/locomotion mode of the user and then automatically adjust the controllers to provide the desired assistance.

The muscle synergy hypothesis [8,9,10,11,12] and the concept of synergy have drawn lots of concerns and been applied to lower limb assistive devices. In neurology, various human motions are considered as combinations of different pairs of joints or synergies that are controlled by the central nervous system [13,14,15]. The nervous system is responsible for coordinating multiple synergies by combining them in a task-specific way. By means of gait synergy, human body symmetry can be transferred into symmetric, rhythmic, and synchronized locomotion, thus achieving gait harmony. Gait synergy (joint coordination during locomotion), including interlimb (between two limbs) and intralimb (within one limb) synergies, has been studied since the 1990s. First suggested by Daffertshofer et al. [16] in 2004, principal component analysis (PCA) is the most commonly used tool to model or derive kinematic synergies [11]. Bockemühl et al. [17] analyzed human catching movement and extracted the first three principal components (PCs), which account for over 97% of the variance. However, PCA’s modeling accuracy on synergy is an issue [18,19,20,21].

Scientists have applied the concept of synergy to the control of assistive devices such as exoskeletons and prostheses [22]. The general idea is to generate appropriate gait trajectories for the affected part from the sound part for different patients based on the synergy. Wearable sensors need to be added to the device or worn by the patient to measure the motion of the sound part. The generated trajectories are in line with the patient’s motion and thus improve human–machine interaction. What’s more, the patient’s motion intention can be deduced from the kinematics of the sound part or residual limb. This idea was first proposed by Vallery et al. and was named complementary limb motion estimation (CLME). Vallery et al. employed PCA to model healthy subjects’ synergy and subsequently generate trajectories for a rehabilitative exoskeleton [18] and an active prosthesis [19]. Their experiments on healthy subjects showed that this method causes less interference on the wearers and encourages participation. However, their estimation results for knee angle were poor. In their later research [20], they proved that BLUE (best linear unbiased estimation) can better model synergy than PCA. Hassan et al. [21] employed PCA and applied synergy to their exoskeleton. Clinical trials on three stroke subjects validated the feasibility of the synergy-based trajectory generation approach. Eslamy et al. [23] also proposed a synergy-based approach for the control of an active knee prosthesis. Gaussian Process Regression was employed to map the input (thigh kinematics) to the output (knee angle). In an intersubject test, average RMSE of 6.36°, MAE of 5.28°, and R^2^ of 0.89 were obtained. Herein, we tried to use a new method (LSTM), other than PCA, to model synergies to reduce the prediction error and improve the results for the control of assistive devices.

On the other hand, disturbed gait synergy is one of the important motor impairments in stroke patients and amputees [24,25,26]. A deeper analysis of patients’ synergies can offer implications for their rehabilitation and assistive device improvement. Continuous relative phase (CRP) is a common mathematical indicator for synergy analysis and quantifying the synergy [27,28,29,30]. Robbins et al. [31] used CRP to examine intralimb synergy in patients with Ehlers Danlos syndrome. Chiu et al. [24] conducted gait experiments on 10 young and 10 elderly subjects to analyze gait synergy; the results indicated that walking speed significantly influenced the CRP results. Combs et al. [32] computed the CRP of groups of stroke subjects before and after locomotion training provided with a weight-supported exoskeleton. However, there are few studies quantifying disturbed synergies in stroke patients and amputees as well as determining the gait kinematics that most affect their synergies.

In this study, we firstly adopted CRP to analyze the synergies in healthy, stroke, and amputee subjects. Their relationship with gait kinematics was explored by stepwise multiple linear regression. Secondly, as a new aspect, we used a long short-term memory neural network (LSTM) to model interlimb and intralimb synergies and compared the results with the simulations by PCA, which is commonly used in the literature. We proposed to model healthy people’s synergy by a better method for generating desired trajectories in line with the wearer’s motion for lower limb assistive devices such as exoskeletons and active prostheses for stroke patients and amputees.

## 2. Materials and Methods

### 2.1. Gait Data Acquisition

The research subjects of this study are healthy people, stroke survivors, and amputees. To analyze and model their gait synergy, the first step is to obtain sufficient gait data. We collected gait data from healthy, amputee and stroke subjects (listed in Table 1). During the experiments, written consent was obtained from each subject.

First, 8 male able-bodied subjects without gait pathology (height: 1.73 ± 0.07 m, weight: 61.6 ± 5.2 kg) were recruited in the healthy group. All subjects wore an IMU-based wearable motion capture system (Perception Neuron, 1.0, Noitom, Beijing, China). The IMU-based system can measure joint angle for gait analysis [33,34,35]. The wearable motion capture system is convenient, with acceptable accuracy as compared to the traditional optical motion capture system [36,37]. Multiple cycles of gait data can be recorded continuously. Seven inertial measurement unit (IMU) nodes were placed on the wearer’s lower limbs by tight straps with Velcro. The acceleration, angular velocity, and quaternion of the lower limb parts and angle of different joints could be obtained from the wearable system. Gait experiments were conducted on level ground (a 19 m walkway). Subjects were instructed to walk at their self-selected speeds. Enough rest was given between two trials. There are 20 trials for each subject.

**Figure 1:**
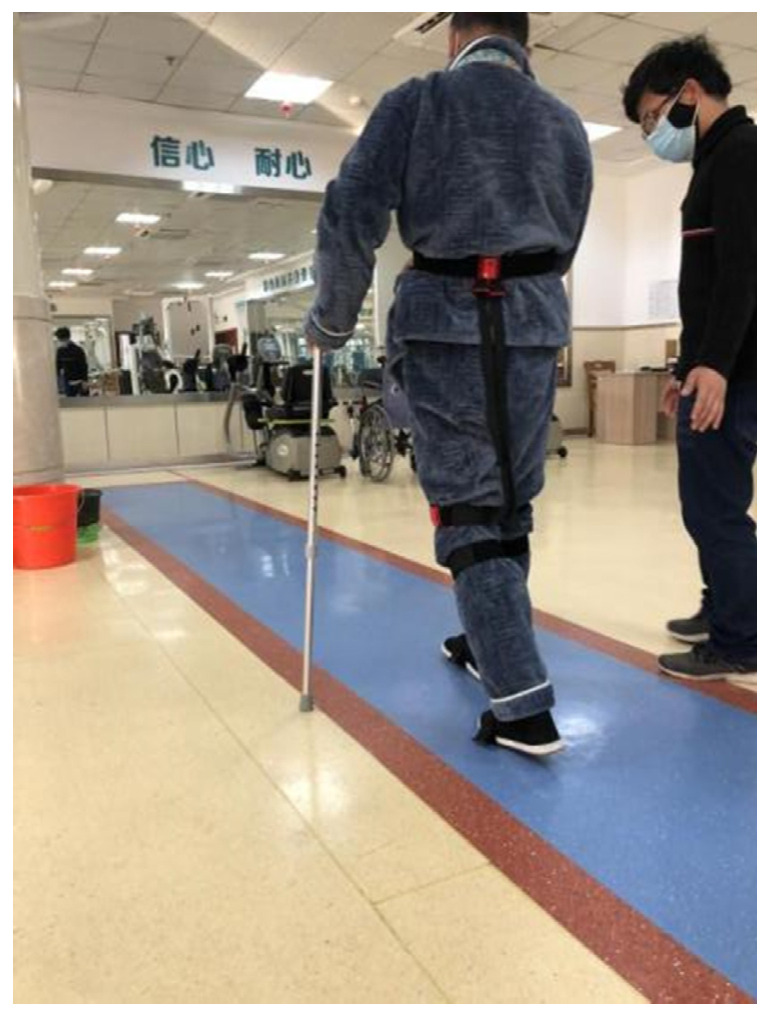
Gait experiments of stroke patients.

Second, 12 stroke subjects were recruited from a hospital (Figure 1) based on the criteria: (1) left or right hemiparesis; (2) over 2 months since onset; and (3) able to walk 12 m without help from others. Similarly, all subjects were asked to walk on a 6 m walkway with the same wearable motion capture system (Noitom^®^ Perception Neuron, 1.0). Considering their leg weakness, they walked 5 trials at their self-selected speeds and enough rest was given between each trial. All of them were informed that they are allowed to leave the walking test any time they wanted. There were 11 subjects (height: 1.66 ± 0.07 m; weight: 49.9 ± 8.5 kg; onset time: 16.3 ± 21.8 mo) who completed the test, since 1 subject quit after the first trial.

Finally, we obtained amputee gait data from 8 unilateral transfemoral amputee subjects (height: 1.75 ± 0.07 m; weight: 70.3 ± 9.1 kg; time since amputation: 10.2 ± 6.3y) from [38]. They all had worn prostheses for over six months. No gait-related diseases were reported by them. Each subject walked on a 10 m path at a self-selected speed.

### 2.2. Synergy Analysis

CRP is adopted to quantify the disturbed interlimb and intralimb synergies of the stroke patients and amputees and then explore the influence of kinematics on the disturbed synergies.

#### 2.2.1. Continuous Relative Phase

CRP is adopted to quantify the disturbed interlimb and intralimb synergies of the stroke patients and amputees and then explore the influence of kinematics on the disturbed synergies. In this study, we use a Hilbert transform to calculate the CRP. The Hilbert transform was suggested by Peters et al. [28] to analyze interlimb and intralimb gait synergies. First, phase angles are calculated based on a measured signal *x*(*t*) and its Hilbert transform *H*(*t*) *= H*(*x*(*t*)):

(1) $$H\left(x\left(t\right)\right)=\frac{1}{\pi }P.V.\int_{-\infty }^{\infty }\frac{x\left(\tau \right)}{t-\tau }d\tau$$

Here, *P.V*. means that the integral is based on the Cauchy principal value. Then the phase angle can be calculated by:

(2) $$\varphi \left(t_{i}\right)=\arctan\left(\frac{H\left(t_{i}\right)}{x\left(t_{i}\right)}\right)$$

The CRP at time *t* CRP_t_ between the two signals *x*_1_(*t*) and *x*_2_(*t*) can be calculated by:

(3) $$\mathrm{CRP}\left(t_{i}\right)=\varphi_{1}\left(t_{i}\right)-\varphi_{2}\left(t_{i}\right)$$

A self-written program based on MATLAB (The Mathworks Inc., Natick, MA, USA) was used for synergy analysis on the data from the able-bodied, stroke and amputated subjects. One representative gait cycle’s joint angles were randomly selected for each subject’s limbs in each trial. Then, the joint angles of one gait cycle were interpolated to 100% for convenience. The data were then curve fitted by the sum of sinusoid functions using the curve-fitting tool in MATLAB. The riding waves and uneven data were eliminated.

The root mean square error (RMSE) and Pearson correlation coefficient (PCC) were used to measure the similarities and correlations of the two CRPs, respectively. The RMSE denotes the deviations or closeness between two curves. The following equation calculates the RMSE between the two CRP values *CRP*_1_ and *CRP*_2_:

(4) $$\mathrm{RMSE}=\sqrt{\frac{\sum_{i=1}^{n}{\left(CRP_{1}\left(t_{i}\right)-CRP_{2}\left(t_{i}\right)\right)}^{2}}{n}}$$

PCC measures the matching degree between two groups of values.

(5) $$\mathrm{PCC}=\frac{\sum_{i=1}^{n}\left(CRP_{1}\left(t_{i}\right)-\bar{CRP_{1}\left(t\right)}\right)\left(CRP_{2}\left(t_{i}\right)-\bar{CRP_{2}\left(t\right)}\right)}{\sqrt{\sum_{i=1}^{n}{\left(CRP_{1}\left(t_{i}\right)-\bar{CRP_{1}\left(t\right)}\right)}^{2}{\left(CRP_{2}\left(t_{i}\right)-\bar{CRP_{2}\left(t\right)}\right)}^{2}}}$$

#### 2.2.2. Decomposition Index

To better analyze interlimb and intralimb synergies, we also adopt the concept of the decomposition index (DI), which is a scale proposed by Bastian et al. [39] to quantify decomposition movements. A decomposition movement occurs when two adjacent or related joints have different movements (one moves while the other pauses). The DI is the percentage of the time of decomposition movement in a gait cycle:

(6) $$\mathrm{DI}=\frac{T_{dec}}{T_{Gait}}$$

In the above equation, *T*_dec_ is the duration of decomposition movement in a gait cycle. *T*_Gait_ refers to the duration of a gait cycle. By definition, when the angular velocity of a joint is less than 5°/s, it is regarded as paused. However, when the angular velocity is higher than 5°/s, it is considered to be moving. For the lower limbs, there are three pairs of joints (hip–knee, knee–ankle and hip–ankle) that have DIs. In this study, the DI of the hip–knee joint is considered to help analyze interlimb and intralimb synergies. Then, the relationships between the DI and different kinds of CRPs were analyzed. Note that the decomposition movement is not considered a physical defect but a compensatory movement for the gait. The changes in the DI reflect subjects’ motion adjustments. It is thus interesting to explore the decomposition movements and DI in both stroke patients and amputees.

### 2.3. Stepwise Regression

Here, we explore the relationship between gait kinematics and synergies in stroke patients and amputees. Stepwise multiple linear regression was performed to identify the determinants of interlimb and intralimb synergies (CRPs). Stepwise regression is a step-by-step process to select independent variables to build a regression model. In each step, a variable is considered to be added or removed from the variables based on the defined criterion. After each iteration, statistical significance was tested. Before stepwise regression, the collinearities among independent variables were diagnosed. The analysis was performed on SPSS v19 from IBM, with a significance level of 0.1. For example, when analyzing gait data of the affected sides of stroke patients, DI_sw_, DI_sta_, percentage, ROM_hip_, ROM_knee_, and speed were selected for collinearity diagnostics.

### 2.4. Synergy Modeling

LSTM was adopted to model the interlimb and intralimb synergies of able-bodied subjects, respectively. Then, we also used PCA, which is commonly used in the literature to model healthy subjects’ different synergies. At last, comparisons were made between the experimental results of LSTM and PCA on the modeling of interlimb and intralimb synergies.

#### 2.4.1. Long Short-Term Memory Neural Network

LSTM networks are commonly used recurrent neural networks. LSTM is popular in recent years for its ability to regain long-term information. For example, LSTM can be employed to classify human activity [40], forecast PM 2.5 [41], and monitor machine health [42]. The following equations give the algorithm for an LSTM model with forget gates:

(7) $$f^{\left(t\right)}=\sigma \left(W_{1}^{f}x^{\left(t\right)}+W_{2}^{f}h^{\left(t-1\right)}+b_{f}\right)$$

(8) $$s^{\left(t\right)}=g^{\left(t\right)}⊙i^{\left(t\right)}+s^{\left(t-1\right)}⊙f^{\left(t\right)}$$

(9) $$i^{\left(t\right)}=\sigma \left(W_{1}^{i}x^{\left(t\right)}+W_{2}^{i}h^{\left(t-1\right)}+b_{i}\right)$$

(10) $$o^{\left(t\right)}=\sigma \left(W_{1}^{o}x^{\left(t\right)}+W_{2}^{o}h^{\left(t-1\right)}+b_{o}\right)$$

(11) $$h^{\left(t\right)}=tanh\left(s^{\left(t\right)}\right)⊙o^{\left(t\right)}$$

where the internal state $s^{\left(t\right)}$ is influenced by the forget gate $f^{\left(t\right)}$, and where (*σ*) stands for sigmoid functions. The internal state *s*^(*t*)^ is influenced by the forget gate *f*. *W*_1_, *W*_2_ and *b* stand for the corresponding weight matrices and bias parameters, respectively. All the parameters in Equations (7)–(10) are determined during multiple training runs.

Interlimb and intralimb synergies, the relationships among the kinematics in the same or different leg, can be regarded as time-series models, thus LSTM is employed to model them.

#### 2.4.2. Interlimb Synergy Modeling

LSTM is employed to model interlimb synergy, the relationship among the kinematics of the two lower limbs. Thus, an LSTM model is built where the angular velocities and angle data of one side’s limb are set as the input and the angle data (knee or hip joint) of the other side’s limb are the output. Inter-subject simulation is conducted to investigate the universality of the modeled “healthy” interlimb synergy. Specifically, for motion data of any randomly selected subject from the 8 healthy subjects, we estimate one side’s knee and hip angles using the contralateral motion data (angles and angular velocities) based on the interlimb synergy modeled from the other 7 subjects. Then, the estimated knee and hip angles of one side are compared with the original data (ground truth). There are 8 turns where each subject’s data become the testing data and the LSTM model is trained by the data of the other 7 subjects. The RMSE, Pearson correlation, R^2^, and Mean Absolute Error (MAE) are adopted to quantify the estimation performance.

To have a fair comparison, we also employ PCA-based linear regression to perform simulations following the steps in [19,43] based on the above-mentioned protocols. Note that we can finally obtain the linear equations (i.e., the interlimb synergy) for each subject’s data between one side’s hip or knee angle and the contralateral motion data. Estimation errors during the 8 turns of the simulations were calculated.

#### 2.4.3. Intralimb Synergy Modeling

LSTM is also employed to model intralimb synergy, the relationship among the kinematics of two joints in the same leg. Previously, Hernandez et al. [44] also used artificial neural networks to model intralimb synergy and then estimated knee angle. However, 5 IMUs were used. In [45], we compared our LSTM-based method with other existing methods that used different types or numbers of sensors and proved to have better estimation performance. Again, we also employed PCA-based linear regression and calculate simulation errors to have a fair comparison.

In LSTM modeling, the original output of one IMU on the wearer’s thigh (angular velocities and accelerations) is set as the input while the wearer’s knee angle data (measured by two IMUs) are the output. Inter-subject simulation is also conducted. We estimate each subject’s knee angle based on the intralimb synergy modeled from the other 7 subjects. Then, the estimated knee angle is compared with the measured knee angle to quantify the estimation performance. Again, there are 8 turns in total.

## 3. Results

### 3.1. Summary of Gait Data

As afore-mentioned, we obtained gait kinematics from stroke, able-bodied, and amputee subjects. Table 2 lists all gait parameters involved in this research (all values are means). There are interlimb and intralimb CRPs quantifying the interlimb and intralimb synergies of three groups of participants. Here, the RMSEs of the CRP values are all marked as CRP for convenience. Note that there are sound sides and affected sides for both the stroke and amputee groups. Interlimb CRP is marked as CRP_(inter)_. The CRP in the stance phase (CRP_st_) and the swing phase (CRP_sw_) are both considered. The percentage stands for the swing phase percentage, the percentage of swing phase of a whole gait cycle.

**Figure 2:**
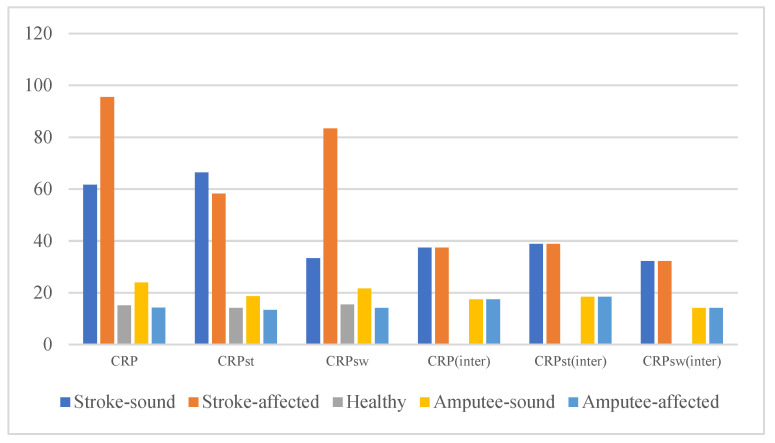
RMSEs of the interlimb and intralimb CRPs of different subjects.

First, from Table 2 and Figure 2, we can find that the RMSEs of the interlimb and intralimb CRPs (synergies) of the stroke group are larger than those of amputee group. The RMSEs of the CRPs in the amputee group are larger than those in the healthy group. Second, the stroke group’s DI is larger than the amputee group’s, while the amputee group’s DI is larger than that of the healthy group. For both the stroke and amputee groups, the DI of the affected or amputated side is larger than the DI of the sound side. Furthermore, both stroke and amputee groups exhibit limited knee and hip range of motions (RoMs). The RoMs of the affected or amputated sides in both stroke and amputee groups are smaller than the sound side.

### 3.2. Synergy Analysis on Stroke and Amputee Subjects

Stepwise multiple linear regression is performed to explore the relationships among gait kinematics and synergies (including interlimb and intralimb synergies) in stroke patients and amputees. Note that only the model with the best regression performance is chosen and shown for each group. Thus, we summarize the factors that have significant influences on interlimb and intralimb synergies (CRPs) of the stroke, healthy, and amputee subjects in Table 3.

First, DIsta is the DI in the stance phase. DIsta is strongly related to the intralimb synergies of the stroke subject’s sound side in the stance phase, those of amputee’s amputated side, and those of healthy subjects. Further, it is strongly related to the interlimb synergy of stroke subjects in the stance phase. On the other hand, DIsw is a determinant of the intralimb synergy of stroke patients’ affected sides and the interlimb synergy of amputees in the swing phase. Compared with healthy subjects, both limbs of the stroke patients and the amputated limbs of the amputees exhibit higher degrees of decomposition movements. This higher decomposition movement significantly influences their interlimb and intralimb synergies. However, amputee’s sound side does not show strong decomposition movement. This may be because amputees and stroke patients perform different compensatory mechanisms to adapt to new interlimb and intralimb synergies, unlike healthy people.

Second, stroke patients and amputees exhibit limited RoMs in hip and knee joints, compared with healthy subjects. ROM_knee_ is a determinant of the stroke patients’ interlimb synergy and intralimb synergy in the stance phase. ROM_hip_ has a strong correlation with the stroke patients’ intralimb synergy in the swing phase. This may be because the stroke patients’ sound-side legs make compensations in the push-off and swing phases [46], so as to restrict the movement of the inverted-pendulum swing. As a result, disturbed synergy was caused. For amputees, the RoMs of the hip and knee have significant impacts on the intralimb synergy of the amputated limb. Thus, we can conclude that limited RoMs in the hip and knee joints in stroke patients and amputees significantly influence their interlimb and intralimb synergies. However, this influence is different in the two groups. For example, stroke patients’ interlimb synergy is mainly influenced by ROM_knee_, while ROM_hip_ influences amputees’ interlimb synergy.

Furthermore, walking speed is a determinant of the intralimb synergy of the sound side and the interlimb synergy for stroke groups. Patients with better CRPs may have greater walking speeds in the tests. This result shows that speed is important in rehabilitation training design for stroke groups.

The above findings can provide useful guidance in rehabilitation training design and assistive device improvement for stroke patients and amputees.

### 3.3. Interlimb Synergy Modeling

#### 3.3.1. Experimental Results of LSTM

LSTM maps the input (kinematics of one side) continuously to the output (contralateral knee and hip angle) based on the interlimb synergy. The model is implemented in Python with 50 training epochs. There are 50 neurons in the hidden layer and one neuron in the output layer.

**Figure 3:**
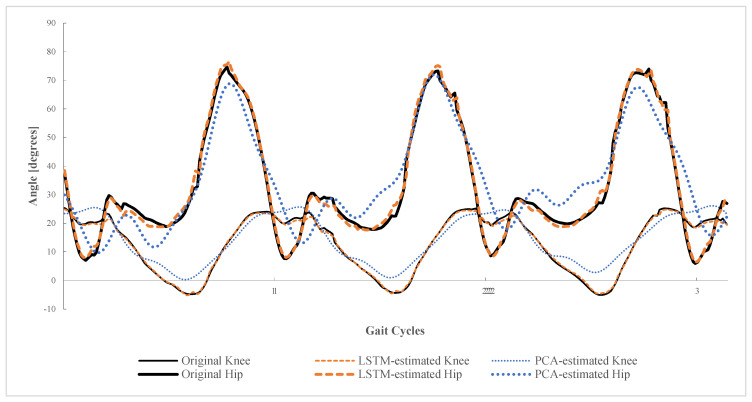
Estimated hip (thicker curves) and knee angles (thinner curves) by PCA (orange curves) and LSTM (blue curves) based on interlimb synergy.

**Figure 4:**
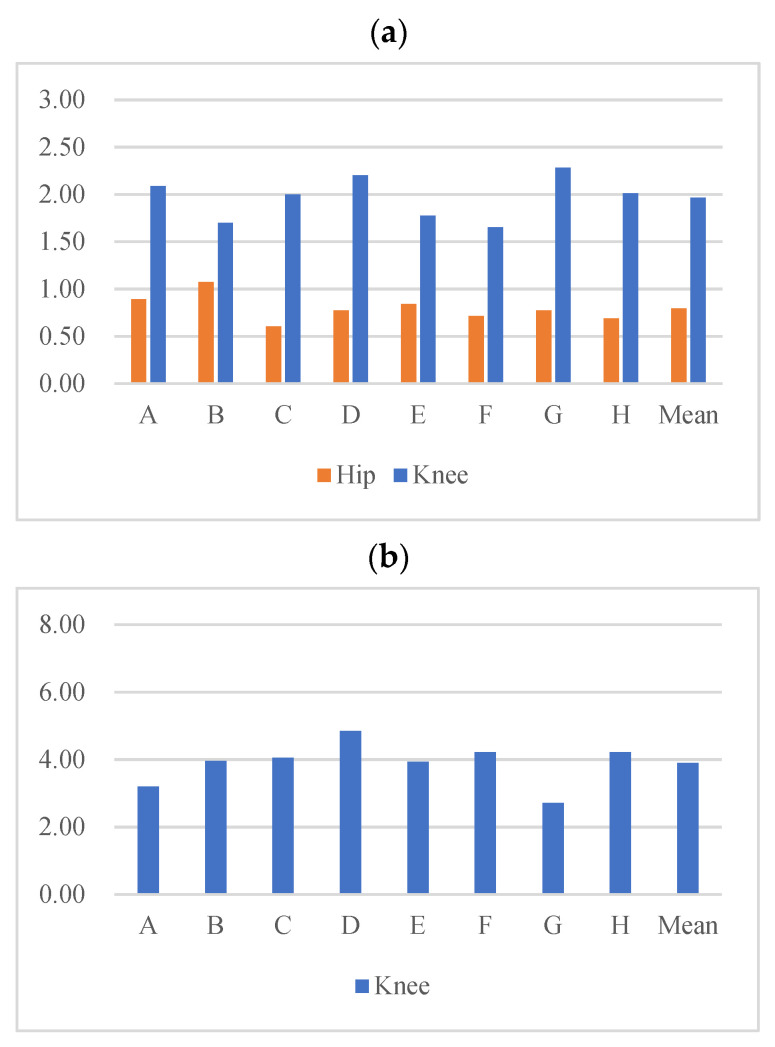
(**a**) RMSE of simulations of hip and knee angles by interlimb LSTM models; (**b**) RMSE of simulations of knee angles by intralimb LSTM models. There is one simulation session for each subject’s data. For example, when estimating subject A’s joint angles, the synergy (interlimb and intralimb) is modeled from the other 7 subjects’ data. “Mean” is the average RMSE of different simulation sessions.

The orange lines in Figure 3 show the estimated knee and hip angles by PCA-based regression in one subject. In each simulation session (eight in total), the RMSE, Pearson correlation, R^2^, and Mean Absolute Error (MAE) are adopted to quantify the estimation performance (average results are given in Table 3). The RMSEs in each session are plotted in Figure 4a. Note that, in each session, one subject’s knee and hip angles are estimated based on the interlimb synergy modeled from the other seven subjects.

The Experimental results in Table 4 indicate that the LSTM model has good estimation performance and modeling on intralimb synergy. The mean RMSE for all simulation sessions is 0.796° and 1.963° for the hip and knee, respectively.

The mean MAEs are 0.632° (hip) and 1.412° (knee). The mean Pearson correlations (0.998 and 0.996) and R^2^ (0.996 and 0.993) are all very close to 1. In Figure 4a, we can find that the RMSEs of different simulation sessions (when one subject’s joint angles are the testing data while the synergy is modeled from the other seven subjects’ data) of the hip and knee angles are close. In addition, the other three scales in all simulation sessions are very close. It can be concluded that the LSTM-based intralimb synergy model has good universality on different subjects.

#### 3.3.2. Experimental Results of PCA

The blue lines in Figure 3 show the estimated knee and hip angles by PCA-based regression in one subject. Note that the interlimb synergy is modeled from the other 7 subjects by PCA. The deviations between the PCA-estimated and original knee and hip trajectories are larger than those between the LSTM-estimated and original ones. For the RMSE, the RMSEs of 5.050° (hip) and 10.353° (knee) by PCA are larger than those of 0.796° (hip) and 1.963° (knee) by the LSTM, respectively (listed in Table 4). Moreover, compared with the results by PCA, the Pearson correlation and R^2^ in simulations by LSTM are much closer to one. It can be concluded that LSTM has better extraction and modeling on interlimb synergy over PCA.

### 3.4. Intralimb Synergy Modeling

LSTM is also adopted to model intralimb synergy. The input of the LSTM model is the thigh kinematics (acceleration and angular velocity) while the output is knee angle. Other LSTM model setting are similar to the interlimb synergy LSTM model.

In the simulation, the RMSE, Pearson correlation, R^2^. and MAE are calculated. The RMSE in each session (when one subject’s knee angles are the testing data while the synergy is modeled from the other seven subjects’ data) is plotted in Figure 4b. In intralimb synergy modeling, the mean RMSE is 3.894° and the mean MAE is 2.193°. The mean Pearson correlation and R^2^ are 0.981 and 0.961, respectively. Compared with the results by PCA (RMSE: 10.312° and MAE: 8.448°, listed in Table 4), the RMSE and MAE results by LSTM are smaller. Additionally, the Pearson correlation and R^2^ in simulations by LSTM are much closer to one. Therefore, the simulation results over the eight subjects’ data indicate that LSTM has smaller simulation errors. Additionally, the Pearson correlation and R^2^ in simulations by LSTM are much closer to one. Therefore, the simulation results over the eight subjects’ data indicate that the intralimb synergy model by LSTM has good universality over different subjects and has better modeling on interlimb synergy over PCA.

## 4. Discussions

In the first study, CRP is used to quantify interlimb and intralimb synergies in stroke patients and amputees. Both stroke patients and amputees have one impaired limb. The impaired limb shows limited hip and knee RoMs. They adopt different interlimb and intralimb synergies or gait patterns, different from healthy people, to compensate for the impaired function of one side. This compensation mechanism adapts them to a higher degree of decomposition movement. Additionally, the limited hip and knee RoMs significantly influence their synergy, but the influence is different in stroke patients and amputees. Stroke patients’ interlimb synergy is mainly influenced by knee ROM while that of the hip influences amputees’ interlimb synergy. This gives us an in-depth view of the motion of stroke patients and amputees. These findings can provide useful guidance in rehabilitation training design and assistive device improvement for stroke patients and amputees.

Second, we tried to find a better method to model synergy. LSTM is adopted to model the interlimb and intralimb synergies of able-bodied subjects. We modeled synergy from able-bodied subjects because we want to use “healthy” synergy in the control of lower limb assistive devices to guide the patients who have disturbed synergy. However, this “healthy” synergy needs to have universality over various subjects. Experiments were conducted for modeling interlimb and intralimb synergy.

In the experiments of interlimb synergy modeling, we also adopt PCA-based regression to model synergy from the same data and then compare the results by LSTM to have a comparison. Experimental results on data from eight subjects indicate that LSTM has better extraction and modeling on interlimb synergy over PCA, and the LSTM model has universality over different subjects. This idea can be applied to the trajectory generation for lower limb exoskeletons that are designed for stroke patients. In future applications, we can first model interlimb synergy from a large group of able-bodied subjects. Then, the trajectory of the rehabilitative exoskeleton (on the paretic side of the wearer) is generated online based on the wearer’s motion data for the sound side and the trained synergy (LSTM model) to encourage the wearer’s active engagement and provide advisable therapeutic effects. This trajectory is adaptive to different wearers and to one wearer’s different gait. This idea is promising for further exploration since engagement is an important factor in stroke rehabilitation.

In the experiments on intralimb synergy modeling, the results indicate that the intralimb synergy model by LSTM has good universality over different subjects. This method can be used to estimate knee angle based on the kinematics of the thigh (on the same side). Additionally, the kinematics of the thigh are measured by only a single IMU. Our results are also compared with those by PCA and prove to enable better synergy modeling over PCA. The intralimb synergy LSTM model can also be applied in lower limb assistive devices, such as transfemoral prostheses. It can help generate a harmonious knee trajectory in line with the amputee’s residual limb’s motion to improve human–machine interaction [18,19]. Moreover, the motion intention of the wearer can be deduced by the residual limb.

## 5. Conclusions

In this study, we analyzed disturbed synergies in stroke patients and amputees for lower limb assistive devices. CRP and LSTM are used to quantify and model interlimb and intralimb synergies, respectively. The relationship among gait kinematics and synergies in stroke patients and amputees has been revealed. First, they perform different compensatory mechanisms to adapt to new interlimb and intralimb synergies, altered from healthy people. The limited hip and knee RoMs significantly influence their different synergies in different ways. Second, LSTM enables better interlimb and intralimb synergy modeling and shows promise for generating trajectories in line with the wearer’s motion and improving human–machine interaction for lower limb assistive devices such as exoskeletons and active prostheses.

## Figures and Tables

**Table 1:** Subject information of stroke, amputee, and healthy groups.

Stroke	Subject	Age (years)	Height (cm)	Weight (kg)	Onset time (months)	Paretic side
	1	35	165	73	48	R
	2	52	159	70	48	R
	3	39	164	85	54	R
	4	55	170	63	5	R
	5	57	155	115	2	L
	6	44	168	85	3	L
	7	53	156	58	9	R
	8	44	170	66	3	R
	9	63	175	69	2	L
	10	57	165	68	4	L
	11	50	175	66	1	R
Amputee	Subject	Age (years)	Height (cm)	Weight (kg)	Amputation time (years)	Amputated side
	1	23	168	60	13	L
	2	30	174	54	1.5	R
	3	24	188	69	18	R
	4	27	169	66	5	L
	5	48	185	82	17	L
	6	32	172	80	7	R
	7	32	170	72	15	L
	8	27	175	78	5	R
Healthy	Subject	Age (years)	Height (cm)	Weight (kg)	BMI (kg/m^2^)	
	A	26	177	62	19.8	
	B	30	165	55	20.2	
	C	26	180	60	18.5	
	D	29	170	69	23.9	
	E	28	175	66	21.6	
	F	31	163	54	20.3	
	G	29	181	64	19.5	
	H	28	174	63	20.8	

**Table 2:** Summary of gait data of different subjects.

	Stroke–Sound	Stroke–Affected	Healthy	Amputee–Sound	Amputee–Affected
CRP	61.64	95.44	15.10	23.97	14.17
CRPst	66.42	58.20	14.15	18.58	13.28
CRPsw	33.33	83.35	15.47	21.63	14.10
CRP (inter)	37.34	37.34	/	17.38	17.38
CRPst (inter)	38.76	38.76	/	18.39	18.39
CRPsw (inter)	32.19	32.19	/	14.13	14.13
Speed (m/s)	0.18	0.18	0.92	/	/
DIst	0.40	0.47	0.21	0.20	0.65
DIsw	0.26	0.34	0.08	0.09	0.21
ROM_knee_ (°)	39.37	35.92	44.38	43.09	42.80
ROM_hip_ (°)	48.72	41.42	62.33	59.52	55.65
Percentage	0.70	0.57	0.53	0.57	0.54

**Table 3:** Stepwise regression results.

Subjects	CRP	Phase	Factors *
Stroke	sound side	whole	speed
		stance	DIsta
		swing	speed
	interlimb	whole	speed
		stance	DIsta
		swing	speed
			ROM_knee_
	affected side	whole	/
		stance	ROM_knee_
		swing	percentage
			ROM_hip_
			DIsw
Amputee	sound-side	whole	percentage
		stance	ROM_knee_
		swing	percentage
	interlimb	whole	/
		stance	/
		swing	ROM_hip_
			DIsw
	amputated side	whole	/
		stance	DIsta
		swing	ROM_knee_
			ROM_hip_
			percentage
Healthy	intralimb	whole	percentage
			DIsta
		stance	percentage
			DIsta
		swing	percentage

Factors *: factors that have the most significant influence.

**Table 4:** Experimental results of LSTM and PCA based on interlimb and intralimb synergies.

Interlimb synergy				
Method	LSTM (hip)	PCA (hip)	LSTM (knee)	PCA (knee)
RMSE (°)	0.796	5.050	1.963	10.353
Pearson (°)	0.998	0.901	0.996	0.868
R^2^	0.996	0.812	0.993	0.761
MAE (°)	0.632	4.109	1.412	8.331
Intralimb synergy				
Method	LSTM (knee)	PCA (knee)		
RMSE (°)	3.894	10.312		
Pearson (°)	0.981	0.835		
R^2^	0.963	0.701		
MAE (°)	2.193	8.448		

## Data Availability

The data that support the findings of this study are available from the corresponding author, W.H.L., upon reasonable request.

## References

[1] Kawamoto H., Lee S., Kanbe S., Sankai Y. Power assist method for HAL-3 using EMG-based feedback controller. Proceedings of the 2003 IEEE International Conference on Systems, Man and Cybernetics.

[2] Jezernik S., Colombo G., Morari M. (2004). Automatic gait-pattern adaptation algorithms for rehabilitation with a 4-DOF robotic orthosis. IEEE Trans. Robot. Autom..

[3] Au S.K., Herr H.M. (2008). Powered ankle-foot prosthesis. IEEE Robot. Autom. Mag..

[4] Sup F., Bohara A., Goldfarb M. (2008). Design and control of a powered transfemoral prosthesis. Int. J. Robot. Res..

[5] Chen B., Zhao X., Ma H., Qin L., Liao W.H. (2017). Design and characterization of a magneto-rheological series elastic actuator for a lower extremity exoskeleton. Smart Mater. Struct..

[6] Ma H., Chen B., Qin L., Liao W.H. (2017). Design and testing of a regenerative magnetorheological actuator for assistive knee braces. Smart Mater. Struct..

[7] Gao F., Liu Y., Liao W.H. (2018). Design of powered ankle-foot prosthesis with nonlinear parallel spring mechanism. J. Mech. Des..

[8] Tresch M.C., Saltiel P., Bizzi E. (1999). The construction of movement by the spinal cord. Nat. Neurosci..

[9] Saltiel P., Wyler-Duda K., d’Avella A., Tresch M.C., Bizzi E. (2001). Muscle synergies encoded within the spinal cord: Evidence from focal intraspinal NMDA iontophoresis in the frog. J. Neurophysiol..

[10] d’Avella A., Saltiel P., Bizzi E. (2003). Combinations of muscle synergies in the construction of a natural motor behavior. Nat. Neurosci..

[11] Ebied A., Kinney-Lang E., Spyrou L., Escudero J. (2018). Evaluation of matrix factorisation approaches for muscle synergy extraction. Med. Eng. Phys..

[12] Celik Y., Stuart S., Woo W.L., Godfrey A. (2021). Gait analysis in neurological populations: Progression in the use of wearables. Med. Eng. Phys..

[13] St-Onge N., Feldman A.G. (2003). Interjoint coordination in lower limbs during different movements in humans. Exp. Brain Res..

[14] Duysens J., van de Crommert H.W. (1998). Neural control of locomotion; Part 1: The central pattern generator from cats to humans. Gait Posture.

[15] Van de Crommert H.W., Mulder T., Duysens J. (1998). Neural control of locomotion: Sensory control of the central pattern generator and its relation to treadmill training. Gait Posture.

[16] Daffertshofer A., Lamoth C.J., Meijer O.G., Beek P.J. (2004). PCA in studying coordination and variability: A tutorial. Clin. Biomech..

[17] Bockemühl T., Troje N.F., Dürr V. (2010). Inter-joint coupling and joint angle synergies of human catching movements. Hum. Mov. Sci..

[18] Vallery H., Buss M. Complementary limb motion estimation based on interjoint coordination using principal components analysis. Proceedings of the 2006 IEEE International Conference on Control Applications.

[19] Vallery H., Burgkart R., Hartmann C., Mitternacht J., Riener R., Buss M. (2011). Complementary limb motion estimation for the control of active knee prostheses. Biomed. Tech..

[20] Vallery H., Van Asseldonk E.H., Buss M., Van Der Kooij H. (2008). Reference trajectory generation for rehabilitation robots: Complementary limb motion estimation. IEEE Trans. Neural Syst. Rehabil. Eng..

[21] Hassan M., Kadone H., Suzuki K., Sankai Y. (2014). Wearable gait measurement system with an instrumented cane for exoskeleton control. Sensors.

[22] Liu K., Xiong C.H., He L., Chen W.B., Huang X.L. (2018). Postural synergy based design of exoskeleton robot replicating human arm reaching movements. Robot. Auton. Syst..

[23] Eslamy M., Oswald F., Schilling A.F. (2020). Estimation of knee angles based on thigh motion: A functional approach and implications for high-level controlling of active prosthetic knees. IEEE Control Syst. Mag..

[24] Chiu S.L., Chou L.S. (2012). Effect of walking speed on inter-joint coordination differs between young and elderly adults. J. Biomech..

[25] Haddad J.M., van Emmerik R.E., Whittlesey S.N., Hamill J. (2006). Adaptations in interlimb and intralimb coordination to asymmetrical loading in human walking. Gait Posture.

[26] Krasovsky T., Levin M.F. (2010). Toward a better understanding of coordination in healthy and poststroke gait. Neurorehabilit. Neural Repair.

[27] Hutin E., Pradon D., Barbier F., Bussel B., Gracies J.M., Roche N. (2012). Walking velocity and lower limb coordination in hemiparesis. Gait Posture.

[28] Peters B.T., Haddad J.M., Heiderscheit B.C., Van Emmerik R.E., Hamill J. (2003). Limitations in the use and interpretation of continuous relative phase. J. Biomech..

[29] Mille R.H., Chang R., Baird J.L., Van Emmerik R.E., Hamill J. (2010). Variability in kinematic coupling assessed by vector coding and continuous relative phase. J. Biomech..

[30] Worster K., Valvano J., Carollo J.J. (2015). Sagittal plane coordination dynamics of typically developing gait. Clin. Biomech..

[31] Robbins S.M., Wolfe R., Chang Y.Y., Lavoie M., Preston E., Hazel E.M. (2021). Inter-segmental coordination amplitude and variability differences during gait in patients with Ehlers-Danlos syndrome and healthy adults. Clin. Biomech..

[32] Combs S.A., Dugan E.L., Ozimek E.N., Curtis A.B. (2013). Bilateral coordination and gait symmetry after body-weight supported treadmill training for persons with chronic stroke. Clin. Biomech..

[33] Seel T., Raisch J., Schauer T. (2014). IMU-based joint angle measurement for gait analysis. Sensors.

[34] Benson L.C., Räisänen A.M., Clermont C.A., Ferber R. (2022). Is this the real life, or is this just laboratory? A scoping review of IMU-based running gait analysis. Sensors.

[35] Prasanth H., Caban M., Keller U., Courtine G., Ijspeert A., Vallery H., Von Zitzewitz J. (2021). Wearable sensor-based real-time gait detection: A systematic review. Sensors.

[36] Robert-Lachaine X., Parent G., Fuentes A., Hagemeister N., Aissaoui R. (2020). Inertial motion capture validation of 3D knee kinematics at various gait speed on the treadmill with a double-pose calibration. Gait Posture.

[37] Noamani A., Nazarahari M., Lewicke J., Vette A.H., Rouhani H. (2020). Validity of using wearable inertial sensors for assessing the dynamics of standing balance. Med. Eng. Phys..

[38] Xu Z., Wong D.W.C., Yan F., Chen T.L.W., Zhang M., Jiang W.T., Fan Y.B. (2020). Lower limb inter-joint coordination of unilateral transfemoral amputees: Implications for adaptation control. Appl. Sci..

[39] Bastian A.J., Martin T.A., Keating J.G., Thach W.T. (1996). Cerebellar ataxia: Abnormal control of interaction torques across multiple joints. J. Neurophysiol..

[40] Steven E.O., Han D.S. (2018). Feature representation and data augmentation for human activity classification based on wearable IMU sensor data using a deep LSTM neural network. Sensors.

[41] Huang C.J., Kuo P.H. (2018). A deep CNN-LSTM model for particulate matter (PM_2.5_) forecasting in smart cities. Sensors.

[42] Zhao R., Yan R., Wang J., Mao K. (2017). Learning to monitor machine health with convolutional bi-directional LSTM networks. Sensors.

[43] Liang F.Y., Zhong C.H., Zhao X., Castro D.L., Chen B., Gao F., Liao W.H. Online adaptive and LSTM-based trajectory generation of lower limb exoskeletons for stroke rehabilitation. Proceedings of the 2018 IEEE International Conference on Robotics and Biomimetics (ROBIO).

[44] Hernandez V., Dadkhah D., Babakeshizadeh V., Kulić D. (2021). Lower body kinematics estimation from wearable sensors for walking and running: A deep learning approach. Gait Posture.

[45] Liang F.Y., Gao F., Liao W.H. (2021). Synergy-based knee angle estimation using kinematics of thigh. Gait Posture.

[46] Raja B., Neptune R.R., Kautz S.A. (2012). Coordination of the non-paretic leg during hemiparetic gait: Expected and novel compensatory patterns. Clin. Biomech..

